# Neonatal Outcome Ascertainment in Mother-Infant Paired Claims

**DOI:** 10.1002/pds.70328

**Published:** 2026-02

**Authors:** Yehua Wang, Nicole E. Smolinski, Thuy N. Thai, Earl J. Morris, Celeste L. Y. Ewig, Sonja A. Rasmussen, Judith C. Maro, Almut G. Winterstein

**Affiliations:** 1 Department of Pharmaceutical Outcomes and Policy, University of Florida, Gainesville, Florida, USA; 2 Department of Population Medicine, Harvard Pilgrim Health Care Institute and Harvard Medical School, Boston, Massachusetts, USA; 3 Department of Genetic Medicine, Johns Hopkins University School of Medicine, Baltimore, Maryland, USA

**Keywords:** claims, measurement, neonatal outcomes

## Abstract

**Purpose:**

Claims data are a valuable source to study neonatal outcomes across a wide range of clinical questions. Infants' delayed enrollment in infant insurance poses challenges in capture of neonatal outcomes, which may be charged to the maternal health plan, posing misclassification risks. We evaluated outcome ascertainment across three infant enrollment scenarios.

**Methods:**

We used Merative MarketScan databases (2012–2018) in the United States to construct a mother-infant linked cohort and assess the outcome ascertaiment precision with varying infant enrollment requirement.

**Result:**

We found that allowing delayed infant enrollment in their own insurance within the first 4 weeks of life retained sample size, nearly doubled case numbers and yielded outcome prevalences similar to those of cohorts with full enrollment since birth. Use of maternal claims in addition to infant claims in this cohort made minor contributions to case capture for neonatal-specific outcomes, while significantly decreasing specificity of more general outcomes. Longer delays in enrollment yielded lower outcome prevalences with higher contributions of maternal claims even for neonatal-specific outcomes. For small for gestational age (SGA), both maternal and infant claims contributed similar proportions of cases.

**Conclusion:**

These findings inform strategies for outcome ascertainment in claims-based perinatal research and emphasize outcome-specific case ascertainment strategies to balance sensitivity and specificity.

## 1 | Background

Health insurance data provide valuable longitudinal information on health outcomes, such as in the assessment of teratogenic effects in linked mother-infant cohorts. Because infants may enroll delayed, insurance companies in the United States allow infant charges under the parental plan(s) [1], posing challenges for accurate measurement of health outcomes. Considering both mothers' and infants' claims is expected to enhance the sensitivity of infant outcome assessment if infant enrollment is delayed, especially in the immediate neonatal period. However, inclusion of maternal claims may lead to misclassification if outcomes that are not specific to neonates and that belong to the mother are mistakenly attributed to the infant. While this risk might be minimal in the assessment of congenital anomalies immediately after birth, the risk of misclassification is greater when assessing generic conditions such as infections.

The Big Data Approaches for Safe Therapeutics in Healthy Pregnancy (BOOST-HP) Project aims for a broad assessment of infant outcomes following fetal exposure to a variety of medications using a combination of data mining and confirmatory pharmacoepidemiologic studies. Evaluated outcomes include some that are not infant-specific. In this brief report, we described an empirical process to support decisions on the inclusion/exclusion of maternal claims to minimize the risk of misclassification of neonatal outcome ascertainment in motherinfant linked claims data studies.

## 2 | Methods

We employed the Merative MarketScan research databases (2012–2018) in the United States to establish a mother-infant linked cohort using a combination of previously established algorithms [2, 3]. To impute an infant's date of birth, we identified the birth year and the month when age (integer variable) changes in the deidentified enrollment files. We then required mothers' delivery date within the same family ID to fall into the imputed birth month. We required mothers to have a minimum of 28 days of continuous enrollment following delivery and created three cohorts based on infants' enrollment: (Cohort 1) full enrollment in the first 28 days, (Cohort 2) enrollment for ≥ 1 but < 28 days in the first 28 days, and (Cohort 3) no enrollment in the first 28 days since birth.

Outcomes were identified using International Classification of Diseases, Versions 9 and 10, Clinical Modification (ICD-9/10-CM) codes in any position in inpatient and outpatient visit claims of both mother and infant within 28 days after delivery for all three cohorts (see Table S1). The outcomes of interest included neonatal seizure/convulsion, pneumonia, small for gestational age (SGA), neonatal jaundice, and neonatal intensive care unit (NICU) admission. SGA included neonatal SGA (e.g., P05.01) and maternal delivery-specific codes (e.g., O36.5990).

We calculated outcome prevalences when ascertained from maternal and/or infant claims for each of the three cohorts. We assumed that cases, that is, individuals with the condition of interest, identified from maternal claims in Cohort 1 were specific to the mother, as infants in this cohort were fully covered by their own health plan. To support this assumption, we conducted a maternal claims record review of pneumonia and convulsion cases (for details, see the Supporting Information).

## 3 | Results

We identified 1 574 268 mother-infant pairs, with 770 659 infants (49%) included in Cohort 1, 598 301 (38%) in Cohort 2, and 205 308 (13%) in Cohort 3. While Cohorts 1 and 2 had similar outcome prevalences (considering both mother and infant claims), estimates for Cohort 3 were lower (Table 1). The median time from estimated birth to enrollment start was 1 day (IQR 0–1) (Figure S1) and from enrollment start to first claim was 5 days (IQR 0–7) (Figure S2). Almost one quarter (24.5%) of infants had their first claim before enrollment (Figure S3).

Outcome capture varied across the evaluated diagnoses and claims sources. In Cohort 1, neonatal-specific conditions were identified mostly by infant claims alone: neonatal seizure (96.1%), neonatal SGA (93.1%), neonatal jaundice (94.1%), and NICU (95.7%). This was different for nonspecific conditions such as convulsion (64.2%) and pneumonia (58.5%), where maternal claims contributed up to 40% of all cases. Through claims record review, we verified that at least 54% of convulsion cases and 64% of pneumonia cases ascertained from maternal claims belonged to mothers (based on claims indicating epilepsy/convulsions or antiepileptic use during pregnancy, and use of solid oral dosage forms of antibiotics or antivirals around the time of pneumonia diagnosis). Only a small proportion of cases included claims for both infants and mothers (e.g., 1.5% of all convulsion cases), further supporting the finding that most cases resulting from maternal claims belong to the mother.

Of note, although neonatal-specific codes are available to denote neonatal seizures, adding more generic convulsion codes increased total case counts by 50% when considering only infant claims. For example, in Cohort 1, using neonatal seizure code only captured 1437 cases, while adding general convulsion codes added an additional 593 cases (Table 1), suggesting that reliance on more infant-specific coding will underestimate prevalences.

Capture of SGA included both infant and maternal claims, with infant-specific SGA coding being prominent on infant claims (93.1%) and delivery-specific SGA coding on maternal claims (~100%). Assuming that all coding captured the current infant and not historic maternal illness, reliance on solely infant claims would have missed 44.0% of all cases in Cohort 1, while reliance on maternal claims alone would have missed 33.8%. Using both mother and infant claims for SGA measurement significantly added case numbers with low risk of misclassifying maternal disease as infant outcome (in Cohort 1, only 5.2% of neonatal SGA cases and 0.1% of maternal SGA cases were identified from mother claims only and infant claims only, respectively) [4]. Other assessed outcomes did not have this gain.

**FIGURE 1 |:**
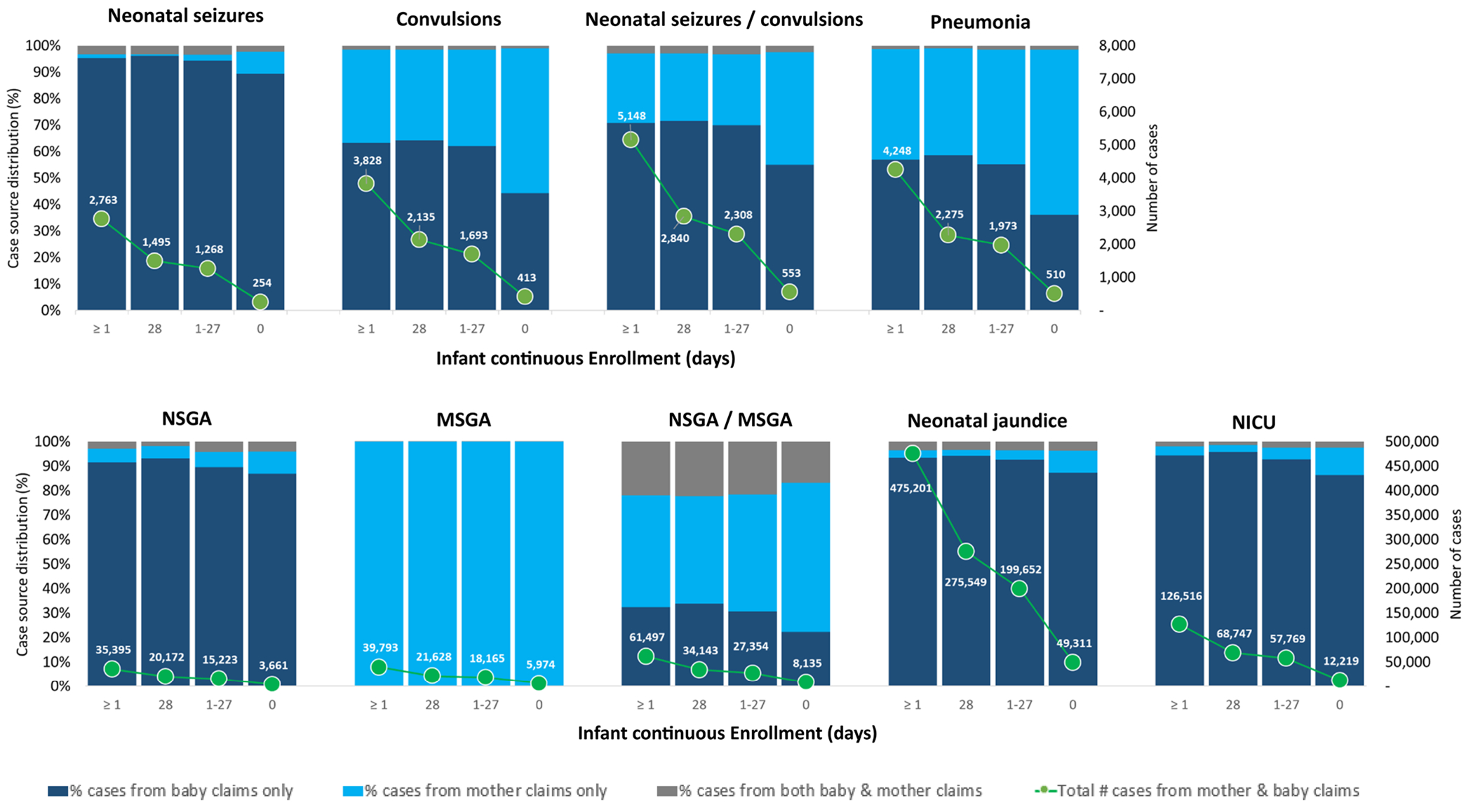
The number of cases and distribution of cases among mother and infant claims. ≥ 1 d: Infant enrolled at least 1 day in the first 28 days (Cohorts 1 and 2); 28-d: 28 days’ full enrollment (Cohort 1); 1–27 d: Delayed infant enrollment but enrolled in the first 28 days (Cohort 2); 0d: No enrollment in the first 28 days (Cohort 3). CE, continuous enrollment; MSGA, maternal (delivery)-specific SGA codes (e.g., O36.5990); NICU, neonatal intensive care unit; NSGA, neonate-specific SGA codes (e.g., P05.01); SGA, small for gestational age.

With the relaxed infant enrollment requirement in Cohort 2, the distribution of cases between infant and maternal claims was similar (Table 1 and Figure 1) to Cohort 1. Consistent with the smaller number of infants in the cohort, the total number of cases for each outcome identified in Cohort 2 was about 80% of those in Cohort 1. If we combined Cohorts 1 and 2, requiring infants to have at least 1 day of enrollment during their first 28 days, sample size and case numbers almost doubled (e.g., 1495 neonatal seizure cases for Cohort 1 only and 2763 cases for Cohort 1 + 2) with similar prevalence estimates and similar distributions of cases contributed by infant and maternal claims (Figure 1).

Comparing Cohort 3 with Cohort 1, outcome prevalences dropped significantly. This drop was least pronounced for maternal SGA and pneumonia, for which most cases were contributed by maternal claims. As expected, the proportion of nonneonatal-specific outcomes identified in maternal claims increased significantly when compared to Cohort 1 and Cohort 2. For neonatal-specific conditions, prevalences dropped about 30% between Cohort 1 and Cohort 3 (e.g., neonatal seizure, 0.19% in Cohort 1 vs. 0.12% in Cohort 3), but the proportion of cases contributed by maternal claims increased (e.g., neonatal seizure, 0.7% in Cohort 1 vs. 8.3% in Cohort 3).

## 4 | Discussion

In this study, we assessed how the measurement of neonatal outcomes based on infant and maternal claims changes with varying infant enrollment requirements. We observed that requiring full infant enrollment from birth resulted in the loss of half of the sample of newborns. Outcome prevalence was similar when comparing scenarios that included infants with full or delayed enrollment (but within the first 4 weeks) and allowed retention of more than 85% of the original sample. Infants who did not enroll within 4 weeks had lower outcome prevalence, perhaps because their encounters were not completely captured and were charged to the other parent. Of note, we captured some claims before infants were enrolled, suggesting that some reimbursements occurred retroactively [5].

Our mother-infant linkage approach, imputing infants’ date of birth based on the month when age (in years) changes in the enrollment file, forces infant enrollment at some point. Other linkage algorithms require infant claims within 30 days of the delivery date on maternal claims [2], effectively establishing a similar population as our Cohort 2. The consistently smaller prevalences associated with > 4 weeks delayed enrollment suggest reduced outcomes capture that cannot be remedied with maternal claims, which should be considered when reporting frequency estimates. For causal inference studies, researchers should evaluate whether these infants with extended enrollment delay are systematically different from their earlier enrollment counterparts, potentially resulting in differential misclassification and measurement bias. Based on similar case capture distributions and prevalences, we recommend requiring at least 1 day of enrollment for the infant in the first 28 days for optimal case ascertainment as well as larger infant samples.

In terms of claims source selection, for neonatal-specific conditions such as neonatal seizures and neonatal jaundice, using infant claims alone identified most of the cases. Considering the low risk of misclassification, adding maternal claims as an additional source is expected to be a low-risk option to further enhance case capture. For nonneonatal-specific outcomes such as pneumonia and convulsion, including maternal claims appeared to add mostly maternal rather than infant cases and hence sacrificed specificity (with positive predictive values < 50% considering our claims record reviews of cases ascertained from maternal claims), which is a significant concern in outcome measurement in causal inference studies [6]. This was true even in Cohort 3, suggesting that use of maternal claims for nonneonatal specific outcomes may not be recommended in any scenario of infant enrollment. Both mother and infant claims should be considered in SGA identification, regardless of infant enrollment, although formal validation against valid measures of weight and gestational age of both claim types should be conducted.

Our study has several limitations. First, our assessment of maternal claims specificity relies on the assumption that infant- focused services were charged to the infant’s insurance if the infant was enrolled. While this is intuitive, we cannot exclude that some infant services were charged to the mother’s insurance. Second, our findings may not be generalizable to other populations, such as Medicaid, with state-specific differences in the timing of infant and duration of maternal enrollment [7].

## 5 | Conclusion

Infant enrollment timing and maternal/neonatal claims source influence infant outcome ascertainment. Allowing a brief delay in infant enrollment retained sample size without reducing outcome prevalence, whereas a delay longer than 4 weeks reduced case capture and prevalence. The inclusion of maternal claims contributed minimally to case capture for neonatal-specific outcomes while significantly misclassifying maternal outcomes as those of infants when assessing more general outcomes. Outcome ascertainment strategies should therefore be outcome-specific to balance sensitivity and specificity.

### 5.1 | Plain Language Summary

Infants’ healthcare claims may be temporarily charged under their mothers’ health insurance before they are enrolled in their own plan. Including both infant and maternal claims to identify neonatal outcomes can retain sample size, but can also induce misclassification by mistakenly counting mothers’ outcomes as infants’. This study evaluated how to best utilize U.S. insurance claims data to identify adverse infant outcomes, maximizing case capture while minimizing misclassification.

We found that allowing delayed infant enrollment within the first 4 weeks of life will retain significant sample size and yield outcome prevalences that are similar to those of cohorts with full enrollment since birth. Use of maternal claims in addition to infant claims in this cohort made minor contributions to case capture for neonatal-specific outcomes, while substantially adding maternal rather than infant cases when assessing more general outcomes. Longer delays in infant enrollment yielded lower outcome prevalences with higher contributions of maternal claims even for neonatal-specific outcomes. This work helps researchers design studies that use real-world insurance data to monitor the safety of medications during pregnancy.

## Supplementary Material

Supplement

Additional supporting information can be found online in the Supporting Information section. **Data S1:** pds70328-sup-0001-Supinfo.docx.

## Figures and Tables

**TABLE 1 |:** Case counts and prevalences in three cohorts with varying infant enrollment requirement.

Conditions	Prevalence	*N* (%) of cases based on infant claims only	*N* (%) of cases based on mother claims only	*N* (%) of cases based on infant and mother claims	Total *N* of cases
*Cohort 1: Infant with full enrollment in the first 28 days of life (total eligible pairs N = 770 659)*					
Neonatal seizure	0.19%	1437 (96.1%)	10 (0.7%)	48 (3.2%)	1495
Convulsion	0.28%	1371 (64.2%)	733 (34.3%)	31 (1.5%)	2135
Neonatal seizure/convulsion	0.37%	2030 (71.5%)	728 (25.6%)	82 (2.9%)	2840
Neonatal SGA	0.30%	18 771 (93.1%)	1053 (5.2%)	348 (1.7%)	20 172
Maternal delivery claims SGA	4.43%	14 (0.1%)	21 592 (99.8%)	22 (0.1%)	21 628
SGA	2.62%	11 526 (33.8%)	15 011 (44.0%)	7606 (22.3%)	34 143
Pneumonia	2.81%	1332 (58.5%)	919 (40.4%)	24 (1.1%)	2275
Neonatal jaundice	35.75%	259 196 (94.1%)	6908 (2.5%)	9445 (3.4%)	275 549
NICU admission	8.92%	65 820 (95.7%)	1876 (2.7%)	1051 (1.5%)	68 747
*Cohort 2: Infants with 1–27 days enrollment since birth (N = 598 301)*					
Neonatal seizure	0.21%	1196 (94.3%)	28 (2.2%)	44 (3.5%)	1268
Convulsion	0.28%	1050 (62.0%)	618 (36.5%)	25 (1.5%)	1693
Neonatal seizure/convulsion	0.39%	1615 (70.0%)	619 (26.8%)	74 (3.2%)	2308
Neonatal SGA	0.33%	13 618 (89.5%)	954 (6.3%)	651 (4.3%)	15 223
Maternal delivery claims SGA	4.57%	11 (0.1%)	18 140 (99.9%)	14 (0.1%)	18 165
SGA	2.54%	8369 (30.6%)	13 072 (47.8%)	5913 (21.6%)	27 354
Pneumonia	3.04%	1089 (55.2%)	853 (43.2%)	31 (1.6%)	1973
Neonatal jaundice	33.37%	184 804 (92.6%)	7736 (3.9%)	7112 (3.6%)	199 652
NICU admission	9.66%	53 598 (92.8%)	2747 (4.8%)	1424 (2.5%)	57 769
*Cohort 3: Infants with no enrollment in the first 28 days of life (N = 205 308)*					
Neonatal seizure	0.12%	227 (89.4%)	21 (8.3%)	6 (2.4%)	254
Convulsion	0.20%	183 (44.3%)	226 (54.7%)	4 (1.0%)	413
Neonatal seizure/convulsion	0.27%	304 (55.0%)	235 (42.5%)	14 (2.5%)	553
Neonatal SGA	0.25%	3181 (86.9%)	332 (9.1%)	148 (4.0%)	3661
Maternal delivery claims SGA	3.96%	2 (0.0%)	5965 (99.8%)	7 (0.1%)	5974
SGA	1.78%	1819 (22.4%)	4949 (60.8%)	1367 (16.8%)	8135
Pneumonia	2.91%	184 (36.1%)	318 (62.4%)	8 (1.6%)	510
Neonatal jaundice	24.02%	43 042 (87.3%)	4449 (9.0%)	1820 (3.7%)	49 311
NICU admission	5.95%	10 556 (86.4%)	1361 (11.1%)	302 (2.5%)	12 219

*Note:* All conditions are supposed to be infant outcomes. Neonatal-specific outcomes refer to the outcomes that can happen only to infants such as neonatal seizure, SGA, neonatal jaundice, and NICU admission. Nonneonatal specific outcomes refer to the outcomes that can happen to both mothers and infants such as convulsion and pneumonia.

All mothers in the mother-infant pairs are required to have a full 28-day enrollment since delivery; prevalence was calculated by the total number of cases divided by the total number of eligible mother-baby pairs in that cohort; SGA includes neonatal SGA identified via ICD codes that originated in the neonatal period (e.g., P05.01) and originated from maternal delivery, including maternal delivery-specific codes (e.g., O36.5990).

Abbreviations: NICU, neonatal intensive care unit; SGA, small for gestational age.

## Summary

- Infant enrollment in their own health insurance might be delayed, posing challenges for claims data analyses of infant outcomes.
- Comparing differences in delays, we found that allowing enrollment within the first 4 weeks of life will retain significant sample size and yield similar outcomes prevalences than forcing enrollment at birth.
- Use of maternal claims in addition to infant claims in this cohort made minor contributions to case capture for neonatal-specific outcomes, while significantly misclassifying maternal outcomes as the infant’s when assessing more general outcomes.
- Longer delays in infant enrollment yielded lower prevalences and higher contributions of maternal claims even for neonatal-specific outcomes.
- Case distribution analyses between maternal and infant claims help guide optimal claim source selection for different neonatal outcomes.
