# Supplementary material for: Neonatal Outcome Ascertainment in Mother-Infant Paired Claims
Source: Pharmacoepidemiol Drug Saf. Author manuscript; Available in PMC 2026 Jul 28. (PMC13408236; doi:10.1002/pds.70328)
Supplement: Supplement [file NIHMS2187406-supplement-Supplement.docx]

**Case claims record review**

To further explore whether cases identified from maternal claims are truly infant and not maternal outcomes, we conducted a claims record case review for both convulsion and pneumonia cases originating from maternal claims. We randomly sampled 50 cases of pneumonia and convulsion cases from Cohort 1 (infants were fully enrolled in the first 28 days). Two authors with clinical training (N.E.S. and C.L.Y.E.) reviewed records and determined whether the cases belonged to mothers.

For pneumonia cases, we reviewed diagnoses on medical encounters and pharmacy dispensing claims 7 days before and up to 14 days after the index pneumonia diagnosis. We considered the use of solid oral dosage forms of medications used to treat pneumonia (antibiotics and antivirals) to be indicative of maternal disease.

For convulsion cases, we reviewed diagnoses on medical encounters and pharmacy dispensing claims within 365 days before the delivery date. We considered the presence of seizure and epilepsy diagnoses or prescriptions of antiepileptics to be indicative of maternal disease.


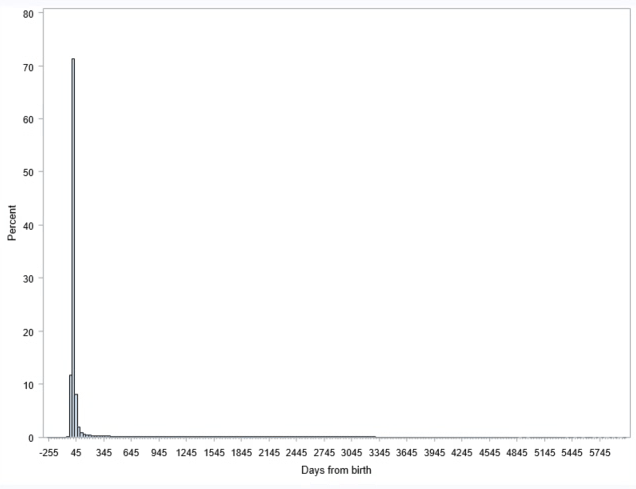


**Figure S1. Time from estimated infant birth to healthcare plan enrollment, in days**


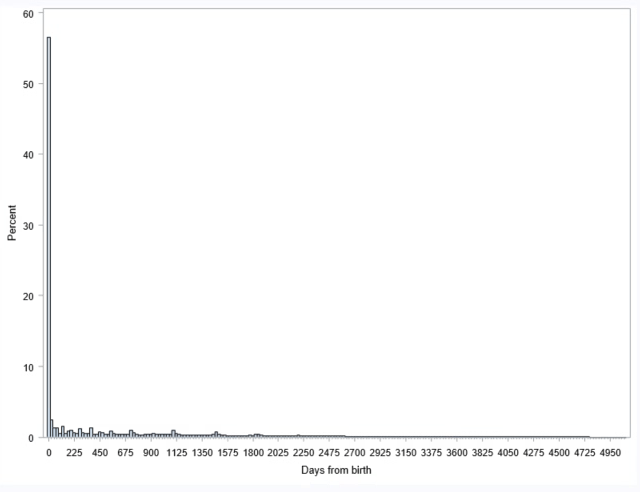


**Figure S2. Time from estimated infant birth to first claim record, in days**


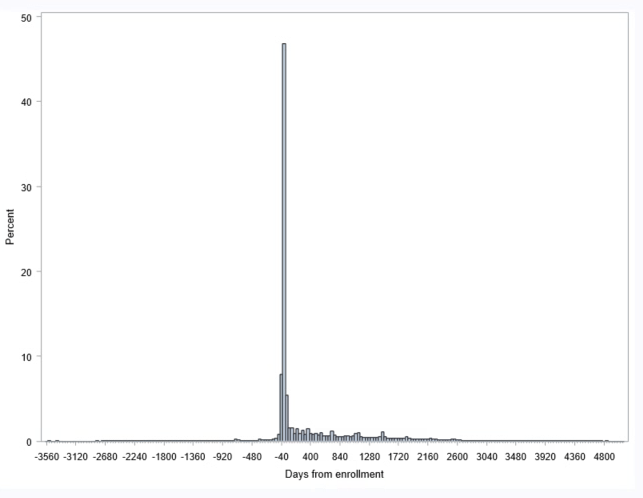


**Figure S3. Time from enrollment to first claim record, in days**

**Table S1. Study outcome value sets**

| **Group** | **Outcomes** | **ICD-9-CM** | **ICD-10-CM** | **CPT** |
| --- | --- | --- | --- | --- |
| **Neonatal specific outcomes** | **Neonatal seizure** | 779.0 | P90 |  |
|  | **Neonatal jaundice** | 774.1, 774.2, 774.30, 774.31, 774.39, 774.4, 774.5, 774.6 | P58.0, P58.1, P58.2, P58.3, P58.41, P58.42, P58.5, P58.8, P58.9, P59.0, P59.1, P59.20, P59.29, P59.3, P59.8, P59.9 |  |
|  | **Neonatal-specific small for gestational age (NSGA)** | 764.00, 764.01, 764.02, 764.03, 764.04, 764.05, 764.06, 764.07, 764.08, 764.09, 764.10, 764.11, 764.12, 764.13, 764.14, 764.15, 764.16, 764.17, 764.18, 764.19, 764.90, 764.91, 764.92, 764.93, 764.94, 764.95, 764.96, 764.97, 764.98, 764.99 | P05.09, P05.10, P05.11, P05.12, P05.13, P05.14, P05.15, P05.16, P05.17, P05.18, P05.19, P05.9, P05.00, P05.01, P05.02, P05.03, P05.04, P05.05, P05.06, P05.07, P05.08 |  |
|  | **Neonatal intensive care unit (NICU)** |  |  | 4168F, 4169F, 99291, 99292, 99295, 99296, 99297, 99468, 99469,  99471, 99472, 99477, 99478, 99479, 99480 |
| **Non-neonatal-specific outcomes** | **Convulsion** | 780.31, 780.32, 780.39 | R56.00, R56.01, R56.9 |  |
|  | **Pneumonia** | 480.0, 480.1, 480.2, 480.3, 480.8, 480.9, 481, 482.0, 482.1, 482.2, 482.30, 482.31, 482.32, 482.39, 482.41, 482.81, 482.82, 482.83, 482.89, 482.90, 483.00, 483.8, 484.3, 482.42, 484.6, 484.7, 485, 486, 487.0 | J12.0, J12.1, J12.2, J12.81, J12.82, J12.89, J12.3, J12.9, J13, J18.1, J15.0, J15.1, J14, J15.2, J15.4, J15.3, J15.211, J15.8, J15.5, J15.6, J15.9, J15.7, J16.8, A37.91, A37.01, A37.11, A37.81, J15.212, B44.0, J17, J18.0, J18.8, J12.9, J10.00, J10.01, J10.08, J11.08 |  |
|  | **Maternal (delivery)-specific small for gestational age (MSGA)** | 656.50, 656.51, 656.53 | O36.512X, O36.513X, O36.519X, O36.591X, O36.592X, O36.593X, O36.599X |  |

Note: Value sets were obtained from previous studies on relevant outcomes. If only available for one ICD era, we crosswalked codes using Codify developed by the American Academy of Professional Coders. The crosswalk was developed based on General Equivalence Mappings (GEMS). An introduction about GEMS can be found here: https://www.cms.gov/medicare/coding/icd10/downloads/icd-10_gem_fact_sheet.pdf

Reference:

Bateman, B. T., Huybrechts, K. F., Maeda, A., Desai, R., Patorno, E., Seely, E. W., ... & Fischer, M. A. (2015). Calcium channel blocker exposure in late pregnancy and the risk of neonatal seizures. Obstetrics & Gynecology, 126(2), 271-278

Wang, X., Wang, Y., Zhu, Y., Montoya-Williams, D., Brown, J., Goodin, A. J., ... & Winterstein, A. G. (2025). Validation of Diagnosis Codes for Low Birth Weight and Small-for-Gestational Age in the Medicaid Analytic Extract Database. *American Journal of Epidemiology*, kwae472.

Yland, J. J., Huybrechts, K. F., Wesselink, A. K., Straub, L., Chiu, Y. H., Seely, E. W., ... & Hernández-Díaz, S. (2024). Perinatal outcomes associated with Metformin use during pregnancy in women with pregestational type 2 diabetes mellitus. *Diabetes care*, *47*(9), 1688-1695.

Thompson, J. L., Kuklina, E. V., Bateman, B. T., Callaghan, W. M., James, A. H., & Grotegut, C. A. (2015). Medical and obstetric outcomes among pregnant women with congenital heart disease. *Obstetrics & Gynecology*, *126*(2), 346-354.
